# A measurement perspective on affirmative action in U.S. medical education

**DOI:** 10.3402/meo.v18i0.20531

**Published:** 2013-04-10

**Authors:** Clarence D. Kreiter

**Affiliations:** Department of Family Medicine, The University of Iowa Carver College of Medicine, Iowa City, IA, USA; Office of Consultation and Research in Medical Education, The University of Iowa Carver College of Medicine, Iowa City, IA, USA

**Keywords:** affirmative action, admission, medical education, selection, bias

## Abstract

**Background:**

The U.S. Supreme Court has recently heard another affirmative action case, and similar programs to promote equitable representation in higher education are being debated and enacted around the world. Understanding the empirical and quantitative research conducted over the last 50 years is important in designing effective and fair initiatives related to affirmative action in medical education. Unfortunately, the quantitative measurement research relevant to affirmative action is poorly documented in the scholarly journals that serve medical education.

**Methods:**

This research organizes and documents the measurement literature relevant to enacting affirmative action within the medical school environment, and should be valuable for informing future actions. It provides summaries of those areas where the research evidence is strong and highlights areas where more research evidence is needed. To structure the presentation, 10 topic areas are identified in the form of research questions.

**Results:**

Measurement evidence related to these questions is reviewed and summarized to provide evidence-based answers.

**Conclusions:**

These answers should provide a useful foundation for making important decisions regarding the use of racial diversity initiatives in medical education.

Medical schools in many parts of the world have enacted or are considering enacting initiatives similar to the affirmative action programs long utilized in the United States (1, 2). The use of affirmative action initiatives to achieve racial diversity at U.S. medical schools has a long history and has been the focus of an ongoing debate in the United States since the Civil Rights Act of 1964 broadened the definition of discrimination to include admissions for educational training (3). Despite the long history of affirmative action in the United States, it remains a disputed legal concept and the U.S. Supreme Court is again hearing arguments and considering briefs from organizations such as the AAMC during the 2012/2013 court session. In medical education, where the competition is particularly intense, the debate tends to elicit strong emotion that is closely tied to an array of political and social issues that divide the nation ideologically. To avoid this controversy, quantitative researchers working within U.S. medical education have been reluctant to offer opinions on socially sensitive research topics related to issues such as group differences and bias that are central to this discussion. Unfortunately, without this expert input, discussions and decisions related to future initiatives designed to attain diversity are unlikely to benefit from the measurement research that has been conducted.

## Introduction

Although measurement studies could inform future and existing implementations of affirmative action initiatives, and perhaps increase consensus, the practical implications of the measurement evidence remains unappreciated to many within the medical education community. Contributing to this problem is the fact that much of the relevant psychometric research is published in journals outside the field of medical education and often employs advanced statistical methods that has in some cases obscured the practical meaning from medical educators unfamiliar with this field of study. This is particularly unfortunate since the measurement concepts of reliability, validity, and bias, although statistically sophisticated, are at their core, scientific definitions of efficiency, consistency, accuracy, and fairness; values which most would agree are primary objectives of the medical school admissions process.

In 2005 the American Education Research Association's Education in the Professions – Division I published an annotated bibliography of the affirmative action research literature relevant to professional education titled: *Affirmative Action and Diversity in Professions Education* (4). With over 200 citations, many targeted towards medical education and advertised as ‘data-driven’, one might conclude that this large body of literature could provide an informative background for facilitating the debate on affirmative action within our medical schools. However, upon closer examination of the research included in this bibliography, it becomes obvious that in many cases where data is used, it is extracted and analyzed to serve an evaluation or advocacy role in support of a position or program. In trying to sort through the research on affirmative action in professional education, it is easy for medical educators to become discouraged by the poor quality of the research methods and the selective presentation of data that is designed to serve an evaluation function. Because the quantitative measurement research addressing key topics in selection for diversity is not adequately documented within the medical education research literature, policy makers within medical education are often unaware of this perspective.

Although the last U.S. Supreme Court rulings on affirmative action (*Gratz et al. v. Bollinger et al*.; *Gruder et al. v. Bollinger et al*.) generated new and as yet unanswered measurement questions related to the educational rationale and impact of diversity, much of the measurement research addressing the key issues related to affirmative action is well established and has produced consistent findings (5, 6). It is unfortunate that despite the maturity of this research, there has been little effort to comprehensively review and interpret the evidence and its implications for diversity in medicine. Current tends both nationally and internationally have heightened the importance of such a summary (1, 2).

The need for such a summary is further indicated by the fact that even within areas where strong measurement-related research evidence exists, opponents in the affirmative action debate continue to make conflicting assertions regarding well-established psychometric measurement facts that are fundamental to understanding and improving affirmative action initiatives. The purpose of this perspective is to logically organize and document the important measurement-related research that is relevant to affirmative action in medical education. The perspective provides summaries of those areas where the research evidence is strong and highlights areas where more research evidence is needed.

To structure this presentation, 10 topic areas are identified in the form of research questions. These questions, when factually answered, should provide an evidence-based foundation for making important decisions regarding the use of racial diversity initiatives in medical education. The ordering of the questions is intended to conform to a logical analysis of the issues that need to be addressed in order to achieve the larger objective of designing a valid evidence-based affirmative action policy in medical education. The selection of questions is necessarily influenced by the anthropological/cultural and legal environment in which medicine and medical education functions in the United States. Although most of the research questions are of importance in application in other societies, different questions might emerge in countries with a different set of demographic, legal, political, ethical, and/or social concerns.

Each of the research questions logically leads to the next, with the first six questions having generated an extensive body of research and relatively conclusive answers. On the other hand, questions 7 through 10 present areas where more research is needed before conclusive evidence-based answers can be obtained. Since the literature is quite extensive, it is not possible in this broad survey of issues to provide a complete and systematic review of the literature for each topic. Rather, this review presents a measurement perspective that primarily utilizes literature reviews and/or meta-analytic summaries that convey the broad consensus and interpretation of the measurement research.

### The 10 research questions

Below is a listing of the 10 measurement research questions:

1. Are the primary measures used in medical school admissions valid and reliable for making selection decisions?
2. Do tests used in the admissions process display racial bias?
3. Do between-group differences in performance on cognitive tests explain the current under-representation of minority groups in U.S. medical education?
4. Can alternate pre-admission measures remediate the underrepresentation of minority groups in medicine?
5. Is it possible to attain racial diversity and proportional representation without large declines in general performance?
6. Do holistic methods represent a psychometrically valid alternative to formulistic methods?
7. Do affirmative action initiatives succeed in graduating competent underrepresented minority physicians?
8. Are there viable selection models that promote diversity while maintaining validity?
9. Do underrepresented minority physicians’ practice choices lead to increased access to care for underserved communities?
10. Does an increase in racial diversity within medical education result in improved educational outcomes?

## The 10 research questions and their evidence-based answers

### Question 1: Are the primary measures used in medical school admissions valid and reliable for making selection decisions?

The predictive power and reliability of the Medical College Admission Test (MCAT), undergraduate Grade Point Average (GPA), and interview-related techniques have been thoroughly investigated and reported. Although variation in criterion reliabilities has produced a wide range of observed validity coefficients, meta-analytic summaries reveal the validity findings to be quite consistent. For example, when validity generalization and meta-analytic methods are used to correct for criterion unreliability and other attenuating influences, undergraduate GPA and MCAT are clearly shown to be useful predictors of both intellectual and clinical performance throughout the medical school years (7). In addition, correlational research examining data from the nation-wide population of U.S. medical students has shown MCAT and undergraduate GPA to predict licensure scores at all levels of training (8). Of the measures used in the admissions process, MCAT is the most reliable and a composite of MCAT and undergraduate GPA is the best predictor of medical school performance (8–10).

The predictive powers of the pre-admission interview techniques have also been extensively examined. Validity coefficients for the traditional interview are very low, and this is at least partially due to poor reliability (9, 11). In response to this finding, new objective structured clinical encounter (OSCE)-style interview formats such as the multiple mini-interview (MMI) were developed to enhance reliability and are now used at a number of medical schools. Research has shown that the MMI can produce a reasonably reliable measure, that is somewhat independent of grades and test scores, for use in the medical school admissions process (12–14). In addition, validity evidence for this new interview-like technique has shown MMI scores to be moderately correlated with pre-admission cognitive measures, national licensure scores, and other medical school outcomes in the pre-clinical and clinical years (13, 15–17).

### Question 2: Do tests used in the admissions process display racial bias?

The question of bias and the statistical techniques used for detecting its occurrence are thoroughly researched topics in the educational measurement literature. The most simple and fundamental measurement-based indication of bias in test scores used for medical school selection is observed when the criterion score predicted from a selection measure consistently produces a prediction that is too low for members of a subgroup. Numerous research investigations of the tests used to select applicants for undergraduate and medical education opportunities report on the predictive power of these tests for majority and underrepresented minority applicants to medical education. The consensus of measurement experts is that this research demonstrates that college admission tests, in general, and the MCAT, in particular, do not exhibit significant bias in prediction (8, 18–21). In addition, using a composite score consisting of undergraduate GPA and MCAT will not under-predict underrepresented minority performance in medical school (20). There is no conclusive research evidence regarding bias in prediction for the MMI and the other interview-type measures used in medicine.

### Question 3: Do between-group differences in performance on cognitive tests explain the current underrepresentation of minority groups in U.S. medical education?

It is a well-established and consistent finding that scores from cognitive ability and educational achievement tests display mean differences by race (22–27). The largest and most socially consequential difference in U.S. medical education relates to the fact that African-Americans score approximately 1.0 standard deviation (SD) below the mean attained by Whites, and that Hispanics fall about 0.70 SD below the majority mean. The most authoritative summaries of this research are by Jensen, Gottfredson, and Linn (28–30). Scores from the MCAT display racial differences very similar to those observed on other educational achievement and cognitive ability tests (20). Although the score distributions for the white, African-American, and Hispanic subgroups have a large overlap, there is extreme disproportional representation at the top of the combined ability distribution. Sackett and Wilk demonstrate this statistical disproportion by showing that with a mean group differential of 1 SD and a selection rate of 0.10 (the top 10%), only 0.01 of the lower scoring group will be selected (31). Because medical schools are highly selective, the size of the observed racial group differences will in practice almost eliminate the selection of certain minorities when that selection is based on maximizing the academic achievement and/or intellectual aptitude measures.

### Question 4: Can alternate pre-admission measures remediate the underrepresentation of minority groups in medicine?

Two basic changes in the measures used for selection have been suggested for reducing mean racial group differences. The first is to modify existing test content. The second suggested change is to supplement the current tests with other types of measures when making selection decisions.

#### Changing the test

The research discussed as part of Question 2 demonstrates that existing tests used for selection are not significantly biased. This implies that any change to existing tests will require more than simply removing some of the existing items that are perceived to be biased. To change the test in an effort to eliminate subgroup differences will require creating new and valid test items that do not exhibit racial subgroup differences. Unfortunately, psychological measurement research has not documented a valid alternate selection instrument or item format that eliminates these subgroup differences. The research shows that all predictive and validated cognitive testing formats display similar mean differences across racial categories, and replacing existing cognitive tests with alternate validated testing formats will not significantly reduce racial subgroup differences (30–32).

#### Using other measures

Another often recommended modification for reducing disproportional selection is for admission offices to supplement existing admission test scores with other measures. Here the psychometric research on the validity and reliability of composite scores generated using weighting equations is relevant. First, in order for a measure to be effective in reducing the underrepresentation of minority populations, the alternate measures must be uncorrelated with, and display a much smaller mean group difference than the cognitive test measures. Sackett and Ellingson demonstrate that a composite may end up increasing group mean differences if the elements of the composite score display a moderately positive correlation (33). In addition, even when the measures are uncorrelated, the reduction in group mean differences is smaller than one might expect. For example, when summing two standardized uncorrelated measures, one with a group mean difference of 1 SD and the other displaying equivalence (0 SD difference), the composite group mean difference is 0.71 SD, not 0.50 SD as one might intuitively expect. Given this psychometric reality, it is not surprising that meta-analytic summaries examining a broad range of alternate measures, including the interview and personality tests, demonstrate that the use of alternate variables in a composite score for admission and selection will not eliminate underrepresentation at the selection ratios used in U.S. medical education (34).

Composite scores with a heavy emphasis on alternate measures exhibit other shortcomings as well. The most salient problem relates to the lack of well-documented alternative predictor variables that are uncorrelated with cognitive measures and related to medical school performance or physician success. To date, research has not identified reliable and valid alternate measures that display low racial group mean differences and also predict performance. In medical school admissions, composite scores that place a heavy emphasis on alternate variables with no predictive validity will produce sub-optimal results in relation to measurable outcomes such as the United States Medical Licensure Examination (USMLE). With a large weight or emphasis on alternate measures, the documented predictive power of both GPA and MCAT will be unnecessarily compromised; resulting in the negative outcome of selecting less successful students from both the over and underrepresented groups (35). For example, since alternate variables such as the interview have been shown to poorly predict medical student performance, outcomes such as the class average on the USMLE will be significantly reduced when using a composite of equally weighted MCAT and interview score compared with using the MCAT alone (9). This point is easily demonstrated statistically, and has also been observed in actual practice (36). Composites scores that include a strong emphasis on measures with low predictive validity will produce lower mean USMLE scores for both the majority and minority groups admitted with that composite measure.

### Question 5: Is it possible to attain racial diversity and proportional representation without large declines in general performance?

To achieve an increase in the number of selected applicants from a racial category that is currently underrepresented, it is usually possible to simply add a positively weighted dichotomous (0, 1) racial category variable in a regression-based equation that otherwise maximizes predicted performance. Alternately, it is possible to maximize mean MCAT scores while implementing constraints on the minimum number of underrepresented minorities desired in a medical class (36, 37). Utilizing these methods to achieve the targeted representations typically sought at U.S. medical schools will not dramatically lower the average class performance. In fact, using either regression-based equations or constrained optimization models will in most circumstances lower the overall class performance only to the degree required to achieve a specified increase in underrepresented minority representation.

Increasing the weighting on a racial category variable to achieve racial diversity in an optimized regression-based selection equation will make the smallest possible trade-off in performance that is required to achieve a specified level of representation of the underrepresented group. Although the weighting of race is a psychometrically valid and reliable method of achieving proportional representation, the U.S. Supreme Court expressly forbids this method. The Court has instead chosen to recommend holistic evaluations as a way to achieve racial diversity (5, 6).

### Question 6: Do holistic methods represent a psychometrically valid alternative to formulistic methods?

Given the problems associated with using composite scores that rely heavily on alternate variables and the legal prohibitions against using psychometrically optimal formulistic methods, attention has again focused on using human judges as a way to improve overall class quality and increase minority representation (5, 6, 38). Unfortunately, a long and well-established line of research already demonstrates that when the goals of the selection process are well defined and operationalized, decisions based on human judges will produce inferior outcomes compared to decisions using statistical methods (39–41). A line of research dating back to 1954 and Paul Meehl's book: *Clinical Versus Statistical Prediction* clearly demonstrates that statistical (actuarial/formulistic) methods based on simple statistical principles work as well or better than holistic ratings for achieving defined selection goals (42). More recently, Hanson et al. compared admission committee performance using holistic methods with independent ratings of component parts of an admission file and found that independent sources of information within the file were lost with holistic ratings (43).

Given that holistic selection has not been shown to be a psychometrically valid alternative to statistical or actuarial-based selection, it seems impossible to logically defend holistic review. However, with the widespread use and recent popularity of holistic review, it seems reasonable to further consider whether there might be some unmeasured and previously unrecognized positive outcome from holistic review. Of course, if one cannot define or measure what is considered to be a positive admissions outcome, it is impossible to conclusively gauge the success of an admissions program. Despite this fact, it is possible to conduct validity studies to scientifically test the assertion that unmeasured goals (e.g., selecting a more ethical or a more sincere group of students) may be achieved through the use of holistic ratings. For example, Kreiter recommends that research should examine the inter-rater reliability of admission committee scores that deviate from regression-based scores (44). Such research would reveal whether admission committees add consistent decision variance beyond that explained by quantitative predictive variables. If there is substantial holistic rater agreement on decisions that deviate from actuarial/statistical decisions, it is possible to maintain that holistic methods contribute positively to the admissions process in some unmeasured fashion. On the other hand, if rater agreement on deviation scores is near zero, this would be conclusive evidence that holistic rating by an admissions committee adds random error rather than rater insight to the selection process. Positive results from this sort of validity research are mandatory for validating holistic selection methods. Unfortunately, to date, there is no positive empirical validity evidence associated with the use of holistic selection.

### Question 7: Do affirmative action initiatives succeed in graduating competent underrepresented minority physicians?

Although the data to answer this question, as it relates to measureable outcomes, exists within medical licensure and certification testing organizations and state sanctioning boards, there has been little statistical reporting that directly addresses the question of underrepresented minority competence. Studies have shown licensure scores for underrepresented minorities to be lower than majority licensure scores; which is predicted by the lower pre-admission scores for this group (20, 45). Only one nation-wide study reports on failure rates on U.S. competency-based tests for licensure. Researchers examined the failure rates from a national sample of all first-time U.S. medical student examinees from 1986 through 1988 and found that approximately 50% of African-American examinees failed the National Board of Medical Examiners (NBME) Part I competency-based licensure test, compared to a 12% failure rate for whites (46). However, this published research failed to convey that a large majority of the failing underrepresented minority examinees do eventually go on to pass licensure exams upon subsequent testing. The ultimate pass-rate for African-Americans on Part I was approximately 90% during the years examined in that study. Another study of five state-run medical schools around the United States found the racial differences between African-American and white pass-rates on the USMLE to be from 9 to 20 percentage points (47). However, that study noted that in the medical schools examined, 10–25% of the African-American students did not take the USMLE, so the percentage pass-rate difference may have been even greater had all students taken the exam.

While the published research is suggestive, a more careful analysis of this data and other licensure data is needed before an evidence-based conclusion can be reached. To definitively answer this research question, researchers within testing organizations should periodically publish failure rates by relevant subgroups for the USMLE licensure exams and all U.S. board exams. Additional research is needed to adequately address competency outcomes related to affirmative action initiatives. Such research should be widely disseminated to inform decision-makers and the public regarding the consequences of affirmative action. The fact that testing organizations are not routinely reporting such data makes it difficult for policy makers to generate informed decisions and leads to further unsubstantiated claims and misinterpretations of poorly sampled data.

### Question 8: Are there viable selection models that promote diversity while maintaining validity?

This important psychometric question has generated little scientific research. The question requires that researchers investigate whether current conceptualizations of measurement validity as it relates to selection can be expanded beyond inferences regarding the individual examinee to include aspects related to class composition. However, determining what characteristics of a medical class are important to medical education outcomes requires further investigation. We do not know the educational effect of class composition in relation to academic majors, gender, ethnicity, race, or SES. Validity-based research is required for establishing whether effective admission selection procedures should include considerations about the overall characteristics of a class (48, 49).

In the case of medical school admissions, mission statements generated for admission programs already contain class-composition objectives, which may logically be used in validity arguments that support diversity objectives. In medical school admissions, the final admit decision is rarely contingent on a single test. It is much more common for multiple sources of data to be used to generate a composite score or global rating that is ultimately used to make the final decision. Whether using formulistic methods or holistic review, the outcome of the data combination process is a ‘yes/no’ decision. It is the final composite measure or the final decision that should be the subject of future validity research. Although the current trend in admissions at many U.S. medical schools is to deemphasize validity research and the evidence it generates, an institution's best chance of successfully attaining a diverse and successful class is to increase an emphasis on research that is aimed towards generating models that simultaneously represent group and individual characteristics in the decision-making framework (49, 50). It seems quite possible that the validity of such models can be established within the context of medical education.

### Question 9: Do underrepresented minority physicians’ practice choices lead to increased access to care for underserved communities?

Whether affirmative action policies can expand health care delivery to underserved communities has been the focus of affirmative action studies since the late 1970s. The studies tend to suggest that underrepresented minority physicians are more likely to serve minority patient populations, low-income populations, and Medicaid recipients (51–54). In addition, there is evidence that race concordance between doctor and patient is preferred by patients and that this concordance leads to greater patient satisfaction (55). No meta-analytic review of this literature exists, and the literature is not sufficiently developed to permit firm conclusions. In addressing the question of whether minority physicians are more likely to serve underserved populations, studies have relied on self-report survey data and the definition of an underserved population has varied. However, many studies do report qualitative evidence suggesting that minority physicians are more likely to provide care for underserved patient populations. Although more research using much stronger methods and ultimately a meta-analytic summary is needed to define the magnitude of this effect, the current literature does tend to suggest that underrepresented minority physicians are more likely to provide care to underserved populations. To draw a more specific conclusion regarding the magnitude of this effect, there is a need for new studies that employ nationwide racially-stratified random samples of physicians and a careful quantification of their practice characteristics.

### Question 10: Does an increase in racial diversity within medical education result in improved educational outcomes?

The U.S. Supreme Court's rationale for affirmative action relies heavily on its assertion that racial diversity has a positive impact on educational quality (5, 6). Although there is currently little convincing evidence to substantiate this, there are lines of research which may ultimately be important for understanding how diversity can impact medical education and medicine. For example, researchers from areas of social psychology, business, and group processes have investigated the impact of diversity on team performance. Cox reviews the team performance literature and concludes that there is evidence that diverse groups are better at problem solving (56). This conclusion is based on results from studies dating back to the 1960s that show group heterogeneity to positively impact the quality of team and group decisions (57–64). There is also some evidence that heterogeneous groups are more creative, and that race may be important in defining heterogeneity (65). Given medicine's increasing reliance on medical teams, these lines of research are likely to be important for documenting the effects of diversity in medicine and medical education. The evidence that relates specifically to the benefits to medical education is very weak and has tended to rely on survey research and has provided only suggestive evidence related to students’ perception of the educational experience (66).

## Concluding remarks

Although the answers to some affirmative action questions await further research, there is strong empirical evidence addressing many of the most important issues. Preadmission measures commonly employed by U.S. medical schools can enable highly efficient selection, and these measures can be used to craft a psychometrically valid selection procedure that optimally manages the performance-diversity trade-off. While it is too early to tell how the revised MCAT2015 will impact the relative standing of currently underrepresented groups, the AAMC organization reasonably expects, based on past history and content analysis, that the new MCAT will not yield substantially different group mean differences (67). One promising line of inquiry is research that expands the application of new measurement and selection models that are sensitive to both individual and class characteristics (35). It is important that the methods used to achieve diversity do not unnecessarily compromise the performance levels of the selected majority students. The impact of diversity on the quality of medical education and medicine will require additional research before evidence-based answers can be provided. While it seems relatively easy to support the U.S. Supreme Court's assertions that the United States has a compelling interest in increasing the numbers of African-American and Hispanic students in U.S. medical schools, this effort must not be achieved at the expense of abandoning the guiding psychometric principles of fairness, efficiency, and equity (5). These principles actually become more important if we truly seek to achieve the goal of diversity.

**Table:** Summary of questions and answers

Questions	Answers
1. Are the primary measures used in medical school admissions valid and reliable for making selection decisions?	Yes
2. Do tests used in the admissions process display racial bias?	No
3. Do between-group differences in performance on cognitive tests explain the underrepresentation of minority groups in medicine?	Yes
4. Can alternate pre-admission measures remediate the underrepresentation of minority groups in medicine?	No
5. Is it possible to attain racial diversity and proportional representation without large declines in general performance?	Yes
6. Do holistic methods represent a psychometrically valid alternative to formulistic methods?	No
7. Do affirmative action initiatives succeed in graduating competent underrepresented minority physicians?	Tentative Yes
8. Are there viable selection models that promote diversity while maintaining validity?	Tentative Yes
9. Do underrepresented minority physicians’ practice choices lead to increased access to care for underserved communities?	Tentative Yes
10. Does an increase in racial diversity within medical education result in improved educational outcomes?	Tentative Yes

Without fully considering the scientific measurement research, it is unlikely that medicine will develop effective and efficient strategies for achieving diversity. Only full and open consideration of the data related to affirmative action policy is likely to be accepted by the public. If scientific methods are used to evaluate the important questions related to affirmative action, we are much more likely to develop policies that serve the greater good. Current trends appear to deemphasize or ignore scientific measurement research and the traditional methods used to establish validity. An approach that ignores the scientific evidence is more likely to fail. Measurement research should be influential in designing diversity initiatives.

## References

[1] Sowell T (2004). Affirmative action around the world: an empirical study.

[2] Romero S (2012). Brazil Enacts Affirmative Action Law for Universities. http://www.nytimes.com/2012/08/31/world/americas/brazil-enacts-affirmative-action-law-for-universities.html.

[3] (1965). Civil rights Act. USC.

[4] Takian A Affirmative Action and Diversity in Professions Education. http://www.aera.net/uploadedFiles/Divisions/Education_in_the_Professions_(I)/Resources/Affirmative%20Action%20Report_March_09.pdf.

[5] *Grutter v. Bollinger* (2003).

[6] *Gruder v. Bollinger* (2003).

[7] Kreiter CD, Kreiter Y (2007). A validity generalization perspective on the ability of undergraduate GPA and the medical college admission test to predict important outcomes. Teach Learn Med.

[8] Julian ER (2005). Validity of the medical college admission test for predicting medical school performance. Acad Med.

[9] Kulatunga-Moruzi C, Norman GR (2002). Validity of admission measures in predicting performance outcomes: the contribution of cognitive and non-cognitive dimensions. Teach Learn Med.

[10] Mitchell KJ (1990). Traditional predictors of performance in medical school. Acad Med.

[11] Kreiter CD, Yin P, Solow C, Brennan RL (2004). Investigating the reliability of the medical school admission interview. Adv Health Sci Educ.

[12] Eva KW, Rosenfeld J, Reiter HI, Norman GR (2004). An admissions OSCE: the multiple mini-interview. Med Educ.

[13] Reiter HI, Eva KW, Rosenfeld J, Norman GR (2007). Multiple mini-interviews predict clerkship and licensing examination performance. Med Educ.

[14] Lemay JF, Lockyer JM, Collin VT, Brownell AKW (2007). Assessment of non-cognitive traits through the admissions multiple mini-interview. Med Educ.

[15] Roberts C, Walton M, Rothnie I, Crossley J, Lyon P, Kumar K (2008). Factors affecting the utility of the multiple mini-interview in selecting candidates for graduate-entry medical school. Med Educ.

[16] Eva KW, Reiter H, Rosenfeld J, Norman GR (2004). The ability of the multiple mini-interview to predict preclerkship performance in medical school. Acad Med.

[17] Eva KW, Reiter HI, Rosenfeld J, Trinh K, Wood TJ, Norman GR (2012). Association between a medical school admission process using the multiple mini-interview and national licensure examination scores. J Am Med Assoc.

[18] Linn RL (1973). Fair test use in selection. Rev Educ Res.

[19] Young JW (2001). Differential validity, differential prediction, and college admission testing: a compehrensive review and analysis.

[20] Koenig JA, Sireci SG, Wiley A (1998). Evaluating the predictive validity of MCAT scores across diverse groups. Acad Med.

[21] Kanazawa S (2012). Intelligence paradox.

[22] Loehlin JC, Sternberg RJ (2000). Group differences in intelligence. Handbook of intelligence.

[23] Hartigan JA, Wigdor AK (1989). Fairness in employment testing.

[24] Jensen AR (1980). Bias in mental testing.

[25] Linn R (1996). Racial and ethnic differences in intelligence in the U.S. on the differential ability scale. Pers Individ Dif.

[26] Jensen AR (1985). The nature of the black–white difference on various psychometric tests: Spearman's hypothesis. Behav Brain Sci.

[27] Roth PL, Bevier CA, Bobko P, Switzer FS, Tyler P (2001). Ethnic group differences in cognitive ability in employment and educational settings: a meta-analysis. Pers Psychol.

[28] Jensen AR (1998). The g factor.

[29] Gottfredson LS (1997). Mainstream science on intelligence: an editorial with 52 signatories, history, and bibliography. Intelligence.

[30] Linn R (2006). Race differences in intelligence.

[31] Sackett PR, Wilk SL (1994). Within-group norming and other forms of score adjustment in preemployment testing. Am Psychol.

[32] Sacket PR, Camara WJ, Kimmel EW (2005). The performance-diversity tradeoff in admissions testing. Choosing students: higher education admission tools for the 21st century.

[33] Sackett PR, Ellingson JE (1997). The effects of forming multi-predictor composites on group differences and adverse impact. Pers Psychol.

[34] Bobko P, Roth PL, Potosky D (1999). Derivation and implications of a meta-analytic matrix incorporating cognitive ability, alternative predictors, and job performance. Pers Psychol.

[35] Kreiter CD, Stansfield B, James PA, Solow C (2003). A model for diversity in admissions: a review of issues and methods and an experimental approach. Teach Learn Med.

[36] Edwards JC, Maldonado FG, Calvin JA (1999). The effects of differently weighted interview scores on the admission of underrepresented minority medical students. Acad Med.

[37] Kreiter CD, Solow C (2002). A statistical technique for the development of an alternate list when using constrained optimization to make admission decisions. Teach Learn Med.

[38] AAMC, Roadmap to diversity: integrating holistic review practices into medical school admission processes (2010). http://services.aamc.org/publications/index.cfm?fuseaction=Product.displayForm&prd_id=294&prv_id=365&cfid=1&cftoken=B2312E42-F8A7-47F4-C33751BE8BA81CBA.

[39] Dawes RM, Faust D, Meehl PE (1989). Clinical versus actuarial judgment. Science.

[40] Grove WM (2005). Clinical versus statistical prediction: the contribution of Paul E. Meehl. J Clin Psychol.

[41] McGaghie WC, Kreiter CD (2005). Holistic versus actuarial student selection. Teach Learn Med.

[42] Meehl PE (1954). Clinical versus statistical prediction.

[43] Hanson MD, Kulasegaram KM, Coombs DL, Herlod J (2012). Admission file review: applying the multiple independent sampling (MIS) methodology. Acad Med.

[44] Kreiter CD (2013). A Proposal for evaluating the validity of holistic-based admission processes. Teach Learn Med.

[45] Vancouver JB, Reinhart MA, Solomon DJ, Haff JJ (1990). Testing for the validity and bias in the use of GPA and MCAT in the selection of medical students. Acad Med.

[46] Dawson B, Iwamoto CK, Ross LP, Nungester RJ, Swanson DB, Volle RL (1994). Performance on the National Board of Medical Examiners part I examination by men and women of different race and ethnicity. J Am Med Assoc.

[47] Lerner R, Nagai AK Racial preferences in medical education: racial and ethnic preferences in admissions at five public medical schools.

[48] American Psychological Association (1999). Standard for educational and psychological testing.

[49] Maeder EM, Wiener RL (2010). Narrowly tailored actuarial models for affirmative action in higher education. Anal Soc Issues Public Policy.

[50] Kreiter CD (2002). The use of constrained optimization to facilitate admission decisions. Acad Med.

[51] Canto JC, Miles EL, Baker LC, Barker DC (1996). Physician service to the underserved: implications for affirmative action in medical education. Inquiry.

[52] Thurmond VB, Kirch DG (1998). Impact of minority physicians on health care. South Med J.

[53] Rabinowitz HK, Diamond JJ, Veloski JJ, Gayle JA (2000). The impact of multiple predictors on generalist physicians’ care of underserved populations. Am J Public Health.

[54] Wayne SJ, Kalishman S, Jerabek RN, Timm C, Cosgrove E (2010). Early predictors of physicians’ practice in medically underserved communities: a 12-Year follow-up study of University of New Mexico School of Medicine Graduates. Acad Med.

[55] Laveist TA, Nuru-Jetter A (2002). Is doctor–patient concordance associated with greater satisfaction with care?. J Health Soc Behav.

[56] Cox TH (1994). Cultural diversity in organizations: theory, research and practice.

[57] Hoffman LR, Maier NR (1961). Quality and acceptance of problem solutions by members of homogeneous and heterogeneous groups. J Abnorm Soc Psychol.

[58] Shaw ME (1981). Group dynamics: the psychology of small group behavior.

[59] McGrath JE (1984). Groups: interaction and performance.

[60] Nemeth CJ (1985). Dissent, group process, and creativity. Adv Group Process.

[61] Nemeth CJ (1986). Differential contributions of majority and minority influences. Psychol Rev.

[62] Nemeth CJ, Wachter J (1983). Creative problem solving as a result of majority versus minority influence. Psychol Rev.

[63] Hong L, Page SE (2001). Problem solving by heterogeneous agents. J Econ Theor.

[64] Hong L, Page SE (2004). Groups of diverse problem solvers can outperform groups of high-ability problem solvers. Proc Natl Acad Sci.

[65] Triandis HC, Hall ER, Ewen RB (1965). Member heterogeneity and dyadic creativity. Hum Relat.

[66] Whitla DK, Orfield G, Silen W, Teperow C, Howard C, Reede J (2003). Educational benefits of diversity in medical school: a survey of students. Acad Med.

[67] (2013). AAMC MCAT 2015 Frequently asked questions. http://www.aamc.org/students/applying/mcat/mcat2015/faqs/.

